# How Thermal Aging Affects Ignition and Combustion Properties of Reactive Al/CuO Nanolaminates: A Joint Theoretical/Experimental Study

**DOI:** 10.3390/nano10102087

**Published:** 2020-10-21

**Authors:** A. Estève, G. Lahiner, B. Julien, S. Vivies, N. Richard, C. Rossi

**Affiliations:** 1 LAAS-CNRS, University of Toulouse, 7 Avenue du colonel Roche, 31077 Toulouse, France; aesteve@laas.fr (A.E.); glahiner@laas.fr (G.L.); bjulien@laas.fr (B.J.); svivies@laas.fr (S.V.); 2 CEA-DAM, DIF, 91297 Arpajon, France; nicolas.richard@cea.fr

**Keywords:** nanothermite, Al, CuO, aging, combustion, initiation

## Abstract

The paper reports a joint experimental/theoretical study on the aging of reactive Al/CuO nanolaminates, investigating both structural modifications and combustion properties of aged systems. We first show theoretically that the long-term storage (over several decades) in ambient temperature marginally affects nanolaminates structural properties with an increase in an interfacial layer of only 0.3 nm after 30 years. Then, we observe that the first thermal aging step occurs after 14 days at 200 °C, which corresponds to the replacement of the natural Al/CuO interfaces by a proper ~11 nm thick amorphous alumina. We show that this aging step does impact the nanolaminates structure, leading, for thin bilayer thicknesses, to a substantial loss of the energetic reservoir: considering a stoichiometric Al/CuO stack, the heat of reaction can be reduced by 6–40% depending on the bilayer thickness ranging from 150 nm (40%) to 1 µm (6%). The impact of such thermal aging (14 days at 200 °C) and interfacial modification on the initiation and combustion properties have been evaluated experimentally and theoretically. Varying Al to CuO ratio of nanolaminates from 1 to 3, we show that ignition time of aged systems does not increase over 10% at initiation power densities superior to 15 W·mm^−2^. In contrast, burn rate can be greatly impacted depending on the bilayer thickness: annealing a stoichiometric nanolaminates with a bilayer thickness of 300 nm at 200 °C for 14 days lowers its burn rate by ~25%, whereas annealing a fuel rich nanolaminates with the same bilayer thickness under the same thermal conditions leads to a burn rate decrease of 20%. When bilayer thickness is greater than 500 nm, the burn rate is not really affected by the thermal aging. Finally, this paper also proposes a time–temperature diagram to perform accelerated thermal aging.

## 1. Introduction

Al-based thermite materials have attracted great attention for decades because of their high energy content and overall stability compared to CHNO energetic mixtures. In addition, ignition and combustion properties, which are easily tuned by modifying the mixture structure and fuel to oxidizer ratio, make these metal-based energetic materials very interesting for many different types of applications: additives in propellants, explosives and pyrotechnics [1,2,3], customized heat sources [4,5], microenergetics [6,7], rapid fuses and microinitiators [8,9,10,11,12,13], brazing of materials, as well as use as a pressure generator as for molecular delivery (such as biological neutralization) [14,15,16]. The potential spreading of nanothermite materials, including reactive thermite nanolaminates [9,17,18,19,20,21,22], in a variety of applications in both defense and civilian domains, absolutely requires a clear statement of how these nanomaterials do behave with time and storage conditions. Despite the active experimental research dedicated to nanothermites over the two last decades, the literature specifically dedicated to aging issues is rare and exclusively concerns nanothermites in the form of mixed powders [23,24]. Wang et al. [24] investigated the short-term storage stability of Al/CuO mixed powder prepared by electrospray method, and observed modifications of the mixture performance after 13 months aging at the ambient temperature. They mentioned a slight lowering of the pressure generation from 686 down to 626 kPa, and an increase in the initiation temperature (from 754 to 775 °C) after observing a slight decrease at mid-term aging. Counter-intuitively, thermal analyses of aged systems indicate a continuous lowering of the onset temperature along storage time. 

It is reasonable to expect that the aging kinetics of sputter-deposited Al/CuO having specific chemical compositions of interfacial layer will qualitatively and quantitatively differ from those of Al/CuO mixed powders with identical stoichiometry, dimensional features, and composition. Along this line, a very recent and thorough experimental study showed that under slow heating rates, the release of gaseous oxygen from the CuO occurring well below reaction onset (i.e., at ~250 °C) initiates the Al oxidation process at the vicinity of native interfaces until a proper Al_2_O_3_ is formed at ~400 °C [25]. Based on these findings, Lahiner et al. proposed a redox reaction model suitable for investigating slow heating rates and low temperature experiments [26]. The model permits describing the kinetics of the reactant and product layer thicknesses (including Al, CuO, Cu_2_O, Al_2_O_3_) of reacting sputter-deposited Al/CuO nanolaminates upon annealing at low temperature (ambient to 500 °C) [26]. This preceding work offers a unique opportunity to investigate Al/CuO nanolaminates aging in an original manner, allowing for efficient in silico screening of structures modification and ensuing performances alteration when the Al/CuO thermite nanolaminates are subject to long-term storage at the ambient temperature and upon annealing as well. 

In this respect, representative types of Al/CuO nanolaminates structures are considered, and their aging analyzed, notably in terms of interfacial layer thickness evolution and energetic reservoir consumption. This aging effect (temperature out of any other chemistry associated with the storage environment) on both initiation time and combustion performances (burn rate) of various structures is then detailed. In complement to simulations, a set of Al/CuO nanolaminates structures was fabricated, and some of them were annealed as prescribed by the proposed aging model to support the theoretical predictions.

This work represents important progress for the community of thermites, not only thanks to the data collected on the aging of Al/CuO nanolaminates, but also as it presents an original methodology that can be applied to other thermite materials. Indeed, whereas the traditional methods in aging investigations are only based on empirical model-free approaches, not supported by physical considerations, our methodology is totally guided by the physico-chemistry of all elementary mechanisms occurring in sputter-deposited Al/CuO multilayers upon annealings. Hence, the elementary mechanism(s) that is (are) activated during an aging process inducing a modification in the thermite performances is known which opens the possibility to the material designers to find solutions in order to remediate. 

## 2. Materials and Methods

### 2.1. Computational Details 

**Figure 1:**
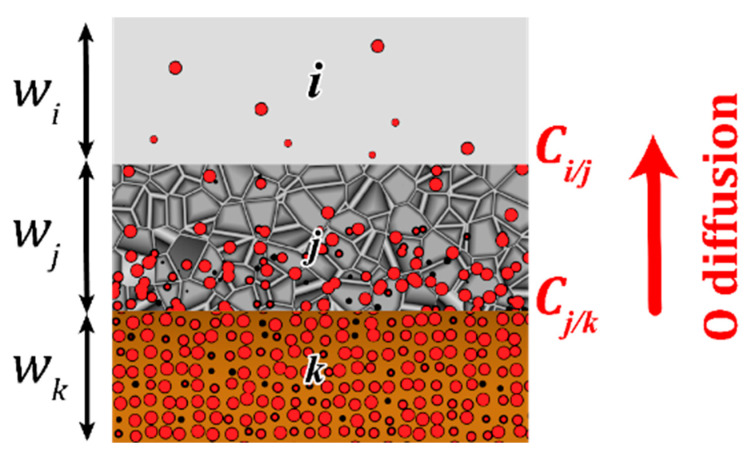
Schematic of the model-system: copper oxide layer *W*_k_ (in orange), alumina barrier layer *W*_j_ (in dark grey) and aluminum *W*_i_ (in light grey). Oxygen diffusion across the interface is schematized with red balls.

A set of elementary diffusion/reaction mechanisms activated at low temperature (oxygen diffusion, structural transformations, and polymorphic phase changes) has been determined by coupling calorimetric characterization with electronic microscopy imaging on Al/CuO nanolaminates [25]. The main finding is that, below Al melting, the rate of reaction is limited by the transport of oxygen species across the growing interfacial layer separating Al and CuO layers. Three main reaction steps were observed to describe the nanolaminate self-heating as depicted in Figure 1: (i) the release of gaseous oxygen from the CuO identified as the first reaction step starting at ~250 °C, this gas accumulates at interfaces, (ii) between 300 and 350 °C, the migration of oxygen species is thermally activated, giving birth to a sudden enhancement of the Al oxidation rate and the subsequent formation of a well-defined amorphous alumina oxide in replacement of the native ill-defined interface. The formation of this amorphous layer hinders further diffusion of oxidizing species, (iii) between 500 and 600 °C, the amorphous alumina turns into a 𝛾-alumina structure that stops further migration of oxygen species. 

Based on these experimental findings, a heterogeneous reaction model was developed, coupling the basic thermal equation with a diffusion/reaction scheme as proposed by Deal and Grove [27]. In brief, for each layer, Al, CuO, Cu_2_O, or Al_2_O_3_, the time evolution of their thicknesses $w_{i}$ is expressed by:

(1) $$\frac{dw_{i}}{dt}=\emptyset \left(t\right)\frac{M_{i}}{3\rho_{i}}$$

$M_{i}$ and $\rho_{i}$ is the molar mass (in kg·mol^−1^) and volumetric density (kg·m^−3^) of the material *i*, *i* representing Al, CuO, Cu_2_O, or Al_2_O_3_. The oxygen flux ∅(t) is defined by the general formulation:

(2) ∅(t)=Dj(C(t)ij−C(t)jk)w(t)j

where $D_{j}$ is the oxygen diffusivity in the material *j* that obeys a Fickian process; *C_i/j,_ C_j/k_* are the oxygen concentrations at interfaces between materials *i* and *j, j* and *k*. *w_i_*, *w_i_, w_k,_* … correspond to the thicknesses of materials *i, j, k,* all representing Al, CuO, Cu_2_O or Al_2_O_3_. The oxygen diffusivity $D_{j}$ follows a single Arrhenius dependence on temperature calibrated from experiments. 

Then, Equation (1) is coupled via a source term related to the energy release during the exothermic reaction.

(3) $$P_{DSC}=\frac{dH\left(t\right)}{dt}-nS\phi \left(t\right)\times Q$$

$P_{DSC}$ is the differential external power (in W) as measured in Differential Scanning Calorymetry (DSC) experiments. The total enthalpy H (in J·g^−1^) is expressed as: $H\left(t\right)=H_{a}+\overset{}{\underset{t,T_{t}>T_{a}}{\sum }}M_{t}h_{t}\theta \left(T\left(t\right)-T_{t}\right)$. With $H_{a}$, the total enthalpy of the bilayer at ambient temperature (*T_a_*) and $h_{t}$ the molar enthalpy corresponding to the various phase transitions *t* that may occur (such as Al melting) at temperature $T_{t}$. $M_{t}$ is the number of moles involved in the phase transition. θ is the Heaviside step function: θ = 0 for *T* < $T_{t}$, and θ = 1 for *T* >$T_{t}$. nSϕ(t)×Q is the source term representing the exothermal Al oxidation, with Q corresponding to the value of the redox reaction 2Al + 3CuO → Al_2_O_3_ + 3Cu given in [28]. For more details about the model implementation, see [26]. Table 1 summarizes the main model parameters used in the following aging study. A more detailed table that includes all main thermodynamic parameters is provided in the Supporting Information file, Table S1.

### 2.2. Nanolaminate Fabrication 

A few configurations of Al/CuO nanolaminates were selected to perform experimental aging. Each sample was characterized by the Al to CuO equivalence ratio ξ, defined as the ratio of the actual Al/CuO ratio to the stoichiometric Al/CuO ratio and bilayer thickness, *w*. Note that in a ξ 1 sample, the aluminum thickness is half the CuO thickness (stoichiometric stack) whereas ξ > 1 corresponds to a fuel rich situation with thicker aluminum layers. The details of the magnetron sputtering of Al/CuO nanolaminates can be found in previous studies (see [30,31]). Throughout the paper, including figures and captions, the following notation was adopted to characterize the Al/CuO nanolaminates: ξ Al/CuO *w*_Al_/*w*_CuO,_ *w*_Al_ and *w*_CuO_ being the Al and CuO layer thicknesses, in nm, respectively.

### 2.3. Experiments

For each produced nanolaminate, thermal analysis, initiation time and burn rate measurements were performed as deposited and after selected aging conditions.

#### 2.3.1. Initiation Time Experiments

The sustained reactions in CuO/Al nanolaminates were initiated via a localized titanium resistive filament, resulting in self-propagating combustion fronts. For the ignition experiments, 700 nm thick Ti film was patterned on glass wafers. Around 12 mm^2^ Al/CuO nanolaminates were sputter-deposited in contact with the Ti filament. The initiation time of the nanolaminates was characterized using a photodiode (Vishay Semiconductors, Heilbronn, Germany, reference BPV10) placed at a few inches distance from the thermite to record the optical emission. 

#### 2.3.2. Burn-Rate Experiments 

Nanolaminates were sputter-deposited along 25 × 2 mm lines, onto a glass substrate. Resistive titanium filaments were patterned at both ends underneath the thermite line to ignite it. The self-propagating reaction velocity, called burn rate, was characterized with a SA3 Photron high-speed camera (West Wycombe, UK) at a framerate of 20,000 fps placed at a few inches distance from the thermite line to record the flame propagation. 

#### 2.3.3. Thermal Analysis

Thermal analyses using a NETZSCH DSC 404 F3 Pegasus (Selb, Germany) were performed under constant heating rates (20 °C·min^−1^) in Ar atmosphere (99.998% pure) at a flow rate of 20 mL·min^−1^. The device, equipped with a DSC-Cp sensor type S and a Platinum furnace, recorded DSC signals in a temperature range from room temperature to 1000 °C. The DSC traces are always normalized by the mass of nanothermite material (around 5 mg).

## 3. Results and Discussion

### 3.1. Modelling the Long-Term Aging at the Ambient Temperature 

The redox model is first used to predict how long-term storage (up to 30 years) at the ambient temperature (300 K) affects the nanolaminate structures and enthalpy of reaction. We perform the simulations on three sets of Al/CuO nanolaminate systems having three different aluminum thicknesses: 75, 100, and 150 nm. CuO thickness is adjusted to obtain a stoichiometric ratio ξ ranging from 1 to 3. Table 2 provides results in term of the energy loss in percentage (EL%) after 30 years of storage at the ambient temperature. EL% is expressed as EL% = $\frac{H_{f}-H_{a}}{H_{a}}$, with $H_{a}$ and $H_{f}$ being the total enthalpy of the bilayer at the ambient temperature after deposition and after the 30 years storage, respectively. Note that the number of bilayers does not affect the results.

We observe that, after a 30 years storage, the EL% remains negligible (<0.1%) clearly demonstrating that Al/CuO nanolaminates are stable for decades under the ambient temperature. Note that the unique physico-chemical process activated at the ambient temperature is the growth of an amorphous alumina layer in the immediate contact of the as deposited natural interface, a process that only consumes a negligible part of the Al reservoir. What is herein called “natural interfaces” corresponds to the intermixed layers formed between sputtered Al and CuO during the deposition. The interface formed upon the deposition of CuO onto Al is a flat Cu_x_Al_y_O_z_ layer (~4 nm in thickness), whereas the interface formed upon the deposition of CuO onto Al is ill-defined over a thickness of ~5 ± 5 nm, as detailed in [25]. For simplification in the model, we took equivalent CuO/Al and Al/CuO interfacial layers: 4 nm of Al_x_Cu_y_O_z_, see Table 1. 

Typically, after 30 years at the ambient temperature, the interface grows marginally (sub nanometer range, not observable experimentally) for all considered systems, even though the results qualitatively show a tendency for the nanolaminates to be more sensitive to aging when considering thin bilayer thicknesses and fuel rich stoichiometry. 

**Figure 2:**
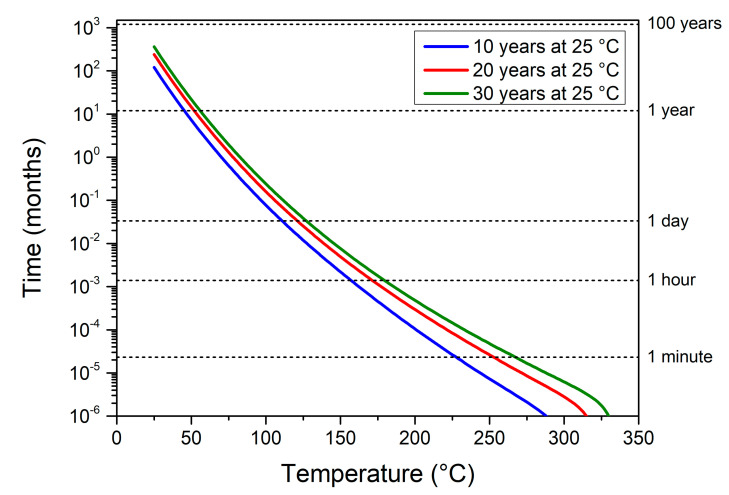
Time–temperature couples giving equivalence to 10, 20, and 30 years natural aging at the ambient temperature for a fuel rich ξ 2 Al/CuO 150/150.

Storing a nanolaminate for long periods of time, such as 10, 20, and 30 years, to investigate the performance evolution is not reasonable. Therefore, thermal accelerated aging experiments are the most usual way to investigate long periods of natural aging. Next, we calculate the temperature–time couple that is equivalent to aging at the ambient temperature for 10, 20, and 30 years. For that purpose, we have adopted an equivalence in terms of the energy loss EL%, i.e., we systematically calculated and identified time–temperature couples leading to the energy loss associated with 10, 20, and 30 years of aging at the ambient temperature, respectively. Figure 2 plots the obtained curves for a fuel rich material corresponding to ξ 2 Al/CuO 150/150 sample. Results show that, for the shortest duration (10 years at the ambient temperature), even though the EL% is nearly zero, a constant temperature of roughly 100 °C applied for one day is required to achieve the EL% equivalence. For 30 years aging at the ambient, applying a 100 °C temperature requires about a week duration to obtain the EL% equivalence. This illustrates the model capability to anticipate any accelerated aging protocol. Note that, as the interfacial layer growth is almost zero, the aging diagram such as the one presented in Figure 2 will not vary when modifying stoichiometry or bilayer thickness. 

### 3.2. Accelerated Thermal Aging

**Figure 3:**
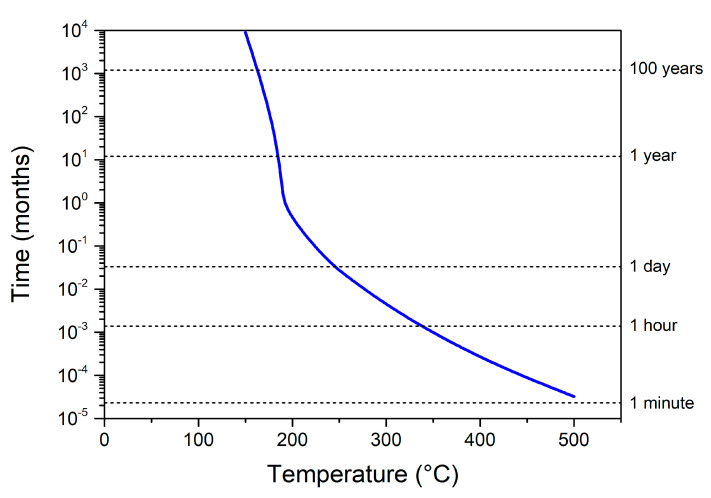
Simulated aging predictions of Al/CuO nanolaminates, depending on time and temperature, to reach an aging leading to the complete conversion of the natural Al_x_Cu_y_O_z_ interface into a well-defined amorphous alumina layer.

Here, as a first aging step, we consider the activation of the mechanism, exhibiting the lower energy barrier, which was introduced in the Table 1 and labelled as Oxygen diffusion through natural Al_x_Cu_y_O_z_. The complete activation of this mechanism corresponds to the replacement of the native ill-defined interface by an amorphous alumina barrier layer that further stops the oxygen transport until ~500 °C [25]. In Figure 3, we plot the couple annealing duration/temperature needed to fully activate the first aging process i.e., the loss of the first DSC peak after aging. We observe two regimes apart from a transition couple: 200 °C/14 days. Below 200 °C, the curve is almost a vertical line with respect to the time plotted in a logarithmic scale. This indicates that temperature is no longer affecting aging time that quickly expands from a few tens of years to centuries. In contrast, above 200 °C, the slope reduces drastically, indicating that the annealing time quickly drops with increasing temperature. Above 350 °C, the time to replace the natural interface by an amorphous alumina falls below the hour duration. 

Interestingly, 200 °C corresponds to the activation of the release of gaseous oxygen from the columnar CuO layer, starting its decomposition. Below 200 °C, the effect of temperature is therefore negligible on nanolaminate properties, as no gaseous oxygen is available in the structure. Note that, for all systems considered in this study, in terms of stoichiometric ratio and bilayer thicknesses, the aging time–temperature diagram will be equivalent to the one shown in Figure 3. This is due to the kinetics of thermal growth: the modification of the interfacial region is equivalent whatever the stoichiometry and bilayer thickness, as soon as the nanolaminate is deposited following the same sputtering conditions.

**Figure 4:**
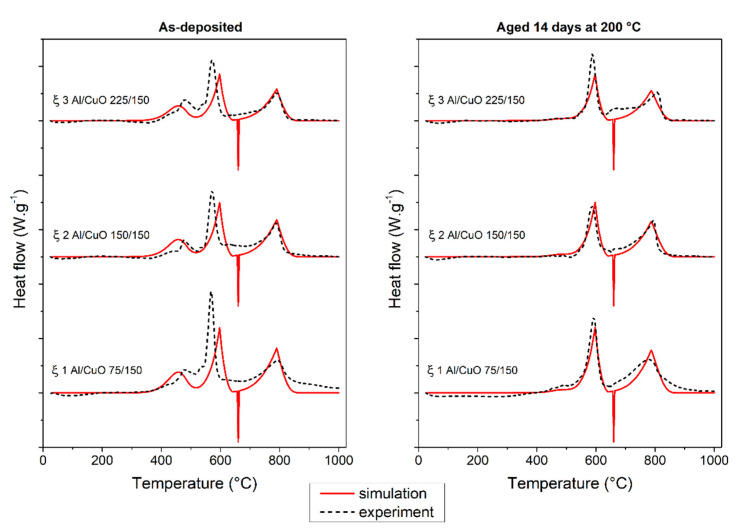
Experimental and theoretical DSC curves of Al/CuO nanolaminates: (left column) as-deposited and (right column) being aged 14 days at 200 °C.

Seeking for an experimental validation of the aging process, we selected the time–temperature aging couple corresponding to the transition point, i.e., sample annealed at 200 °C for 14 days. Figure 4 compares experimental and simulated DSC curves obtained for stoichiometric and fuel rich Al/CuO nanolaminates collected at 20 °C·min^−1^ after deposition (as-deposited) and after being annealed at 200 °C for 14 days (aged). Overall, the simulated DSC results show good agreement with the experimental curves with the three main reaction steps at 440, 578, and close to 800 °C. The first experimental exothermal event between 350 and 540 °C, corresponding to the oxygen diffusion through the native interfaces, is broad showing a two peaks contribution. This is probably due to the difference in nature and thickness of the two natural interfacial layers associated respectively with the deposition of Al onto CuO and CuO onto Al. Instead, the virtual DSC traces feature a single exothermic peak contribution, as we assumed the two Al/CuO and CuO/Al interfaces identical. Most importantly, the first DSC peak totally disappears for both experimental and virtual DSC traces after the annealing at 200 °C for 14 days: this represents a loss of ~20% of the initial energetic reservoir. Note that the model also predicts the theoretical melting of Al is set to 660 °C (endotherm), which is the bulk melting point, whereas it is not always seen experimentally, depending on layer thicknesses, stoichiometry, and heating ramp.

### 3.3. Modelling the Structural Modifications and Associated Loss of Energy Reservoir Upon Thermal Aging

**Figure 5:**
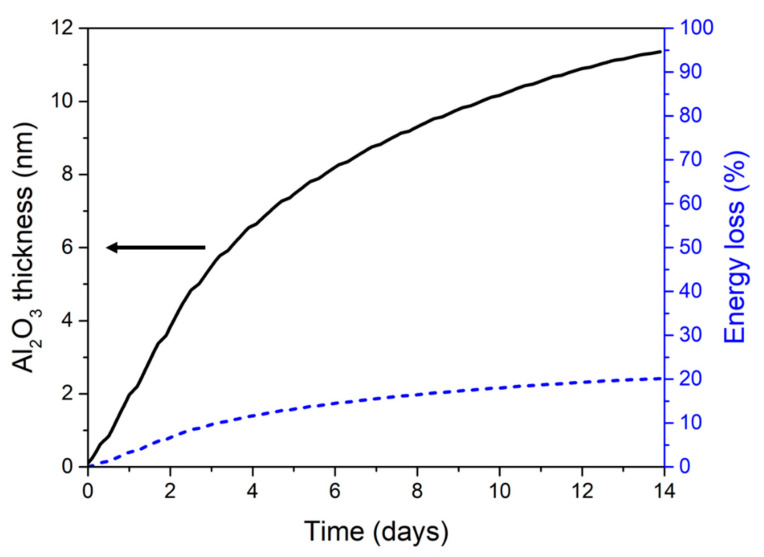
Time evolution of both the amorphous alumina thickness (black line) and Energy loss EL% (dashed blue line), for a ξ 2 Al/CuO 150/150 nanolaminate, aged 14 days at 200 °C.

The structural changes due to thermal aging, when modifying stoichiometric ratio and bilayer thicknesses, may impact the Al to oxygen ratio of aged materials. The kinetics of these changes are theoretically examined for different bilayer thicknesses (*w* = 150–375 nm) and stoichiometric ratio (ξ 1–3). Considering a ξ 2 Al/CuO 150/150 nanolaminates at 200 °C, Figure 5 shows the prediction of the interfacial alumina growth and subsequent energy loss percentage (EL%) evolution with annealing time.

**Figure 6:**
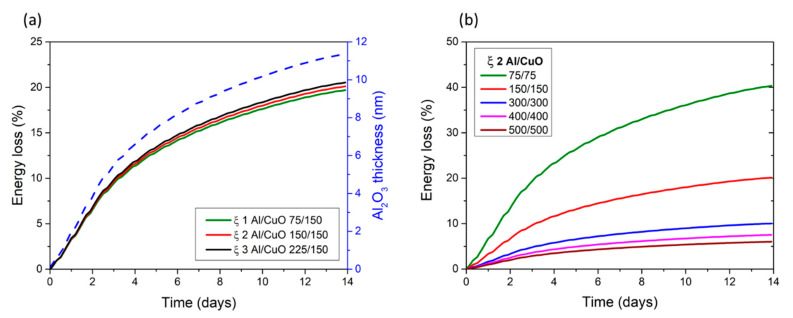
Time evolution of the energy loss EL% upon 200 °C aging conditions for, (**a**) three different stoichiometries (ξ 1–3), (**b**) ξ 2 stack with five bilayer thicknesses *w* = 150, 300, 600, 800, and 1000 nm.

Modelling results show that at 200 °C, a ~11 nm thick amorphous alumina is grown after 14 days leading to an energy loss of 20%. Figure 6a,b shows how the stoichiometry and the bilayer thicknesses affect the energy loss keeping the same aging conditions (200 °C/14 days), respectively. In Figure 6a, the bilayer thickness is kept constant and stoichiometric ratio varies. We observe that EL% decreases when the stoichiometry increases, which is explained by the fact that the reaction enthalpy ($H_{a}$) is maximum at ξ 1 and decreases for fuel rich conditions.

Still considering a fuel rich nanolaminate (ξ 2), we observe a strong variation of the energy loss, from 5 to 40% with the decrease in the bilayer thicknesses (Figure 6b). EL% is of 10 and 8% for bilayer thicknesses of 300 and 400 nm, respectively. This can be explained by the fact that the quantity of Al consumed to grow the amorphous alumina interface remains constant whatever the bilayer thickness, whereas the total quantity of Al increases with the bilayer thickness increase. Hence, increasing the Al quantity in the nanolaminate diminishes the impact of interface evolution and aging. We see that increasing bilayer thickness to the submicron range clearly allows lowering the aging effect, with an energy loss of nearly 6% when reaching *w* = 1 µm. In contrast, when downscaling the bilayers below 300 nm, the effect of aging is impactful, with energy losses largely exceeding 20% of the initial energetic reservoir; a nanolaminate with a bilayer thickness of 150 nm loses 20% of its energetic reservoir after 14 days at 200 °C.

As summary of this first part, we theoretically demonstrated the stability of Al/CuO sputter-deposited nanolaminates at ambient temperature over a few tens of years. We also showed that the full activation of the first aging mechanism, which is the replacement of the native interface by an amorphous alumina, reduces the energy reservoir up to 40% when bilayer thickness is below 300 nm. In order to reduce the impact of interfacial modification upon heating, nanolaminates must be designed with bilayer thicker than 500 nm. In any case, we anticipate that the ignition and reaction performances will be affected as initiation and further reaction are function of the nature and thickness of the layers. This is assessed and discussed in the next section.

### 3.4. Impact of Aging on Nanolaminate Performance

This section discusses the effect of nanolaminate storage on initiation and burn rate. In this section we consider the aged material when its native interface is replaced by the amorphous alumina, which we have shown earlier to require 14 days at 200 °C. Note that all simulations of initiation are performed using the redox model presented in Section 2.1 coupled with heat transport equation to model the propagation. Only the thermal losses and the initiation procedure is different as described in ref. [32]. The multilayer ignition is triggered by imposing a constant heat power density at the left end of the film over a width of 750 µm (details in ref. [32]).

#### 3.4.1. Initiation

**Figure 7:**
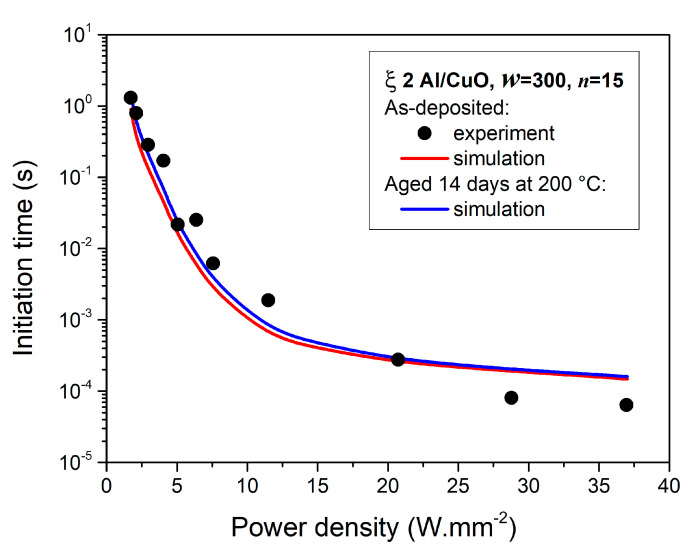
Initiation time as a function of applied power density on a ξ 2 Al/CuO 150/150 nanolaminates composed of black solid circles are experimentally measured for as-deposited samples. The red and blue simulated curves correspond to an as-deposited nanolaminate (red curve) and after being aged 14 days at 200 °C (blue curve), respectively.

Figure 7 plots the initiation time calculated for a ξ 2 Al/CuO 150/150 nanolaminate initiated at different power densities. ξ 2 Al/CuO 150/150 was chosen as this configuration is the widest use in applications related to MEMS based initiators [15]. Overall, the simulation points show good agreement with experimental points. Two regimes can be observed: at low power density, i.e., below 20 W·mm^−2^, the initiation time corresponds to the time needed to heat up the material to its initiation temperature (heating time). Initiation times are in the millisecond to few second range. Above 20 W·mm^−2^, the heating time requested to heat the material to its initiation temperature is very low (sub-millisecond regime). Then the nanolaminate starts reacting until the self-sustained reaction is reached: this reaction time takes from a few µs to hundreds of µs depending on the bilayer thickness. Therefore, above 20 W·mm^2^, the ignition delay is only controlled by the reaction time, explaining the curve flattening at high initiation power densities.

Simulations of initiation time using power densities inferior to 20 W·mm^−2^ better fit the experimental points, while systematic overestimation is obtained beyond this value. This might be explained by the fact that “thermal chocks” may be imposed to the nanolaminate which are not considered in the model, while being observed experimentally [11]. Comparing now aged and non-aged simulation points, initiation is more affected by nanolaminate aging at low power densities (<5 W·mm^−2^): initiation time is 34% longer for aged nanolaminates compared to fresh ones (ξ 2 and *w* = 300 nm). When increasing the power density, this delay drops quickly. Beyond 10–15 W·mm^−2^, the initiation delay is slightly increased by ~7% which can be considered negligible.

Unfortunately it is impossible to characterize the initiation of aged samples because of the concomitant aging of the Ti thin film resistor in contact with nanolaminate.

#### 3.4.2. Burn Rate

**Figure 8:**
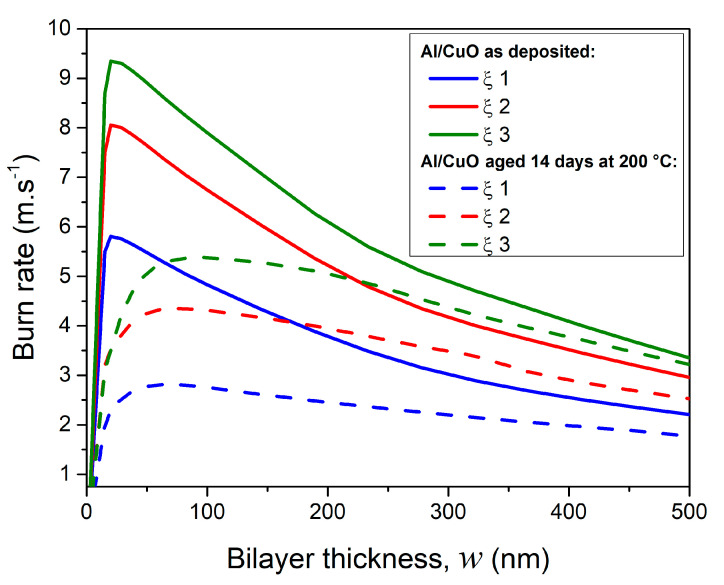
Simulated burn rates of Al/CuO nanolaminates as a function of bilayer thickness for three stoichiometries: ξ 1–3. The total thickness of the nanolaminate as well as heat losses are kept constant by varying the number of bilayers. Solid and dashed curves correspond to as deposited and aged (14 days at 200 °C) nanolaminates, respectively.

To predict the nanolaminates burn rates, we use a home-built propagation model specifically dedicated to the simulation of the self-propagating combustion. This model detailed in [32] has been calibrated empirically to account for high temperature processes driving the flame front propagation. Before exploring how aging affects the burn rate on a vast number of nanolaminate configurations (Figure 8), we start first with a validation step on a reduced set of experimentations (Table 3).

Table 3 assembles a list of unpublished and recently published experiments [31,33] performed on sputter-deposited Al/CuO nanolaminates produced by our team and characterized in combustion. For both experimental and theoretical data, we have considered fresh nanolaminates, i.e., characterized just after sputter deposition, and aged nanolaminates, i.e., being annealed at 200 °C for 14 days.

The first observation is that the model qualitatively reproduces the dependency of the burn rate on dimensions and stoichiometries in studied nanolaminates. Although the model is able to predict the experimental macroscopic burn rate in the ξ 1 Al/CuO 75/150 system (discrepancy between modelled and experimental data ~9%), it fails in predicting the experimental macroscopic burn rate for fuel rich stacks. To explain this, one has to note that the macroscopic burn rate corresponds to the global measurement of the reaction front velocity in the Al/CuO system. As discussed in [33], it corresponds to measuring the microscopic (local) burn rates at the reaction front multiplied by the corrugation of the flame front, which is the ratio of the total geometrical length of the flame to its width. While the exact origin of corrugation is still elusive, it is clearly the result of mechanisms different from the pure condensed phase combustion that fundamentally operates in our propagation model. The corrugation factor is ~1.3 for stoichiometric stacks leading to a macroscopic burn rate close to the microscopic one. However, the flame corrugation factor is of ~3 for fuel rich nanolaminates. The significant corrugation for fuel rich cases leads to a larger burning surface area (× ~3) and, consequently, faster observed burn rate (macroscopic) in fuel rich samples (details in [33]). Hence, considering microscopic burn rate, measured by microscale high speed imaging (not shown in this article but detailed in [33]) and with respect to fresh samples, fair agreement is found with the experimental values, being of 4 and 3.5 m·s^−1^ for ξ 2 and ξ 3 stacks. For aged samples, comparison of fuel rich samples is not straightforward, as the corrugation and microscopic burn rate were not characterized.

As summary, for fuel rich systems, the model is able to predict microscopic burn rate, which is explained by the fact that the model is exclusively based on thermal conduction and does not take into consideration advection and convection mechanisms which both greatly contribute to the flame propagation of fuel rich samples.

Figure 8 plots the burn rates of as-deposited and aged nanolaminates having bilayer thicknesses ranging from 25 to 500 nm and for three different stoichiometric ratios (ξ 1, 2, and 3). Whatever ξ value, aging effect on the burn rate increases with the bilayer thickness decrease. For a bilayer thicknesses inferior to 200 nm, the burn rate is reduced by 30% after an aging at 200 °C/14 days. For bilayer thickness equal to 300 nm, the burn rate drops from 3.4 m·s^−1^ to 2.6 m·s^−1^ and from 4.6 m·s^−1^ to 3.8 m·s^−1^ for ξ = 1 and 2, respectively. For thick bilayer thicknesses (*w* > 500 nm), the burn rate remains unchanged (1 to 2 m·s^−1^ depending on stoichiometry) after aging. This result was expected since the grown oxide at the nanolaminate interface becomes marginal for thick bilayers.

## 4. Conclusions

This paper investigated in detail the long-term storage and thermal aging of Al/CuO sputter deposited nanolaminates. The main findings are that: (1) Al/CuO nanolaminates are stable over decades at the ambient temperature. The energy reservoir is not affected after 30 years of storage under ambient conditions. (2) An aging transition is reached after annealing at 200 °C for 14 days: this corresponds to the replacement of the “ill-defined” Al_x_Cu_y_O_z_ native interfacial layer by an amorphous alumina. This mechanism affects the energetic reservoir and can therefore modify the initiation time and burn rate. (3) The importance of this interfacial modification on the energetic performance (initiation and burn rate) greatly depends on the stack dimensional characteristics, i.e., bilayer thickness and stoichiometric ratio: a Al/CuO stack having a bilayer thickness of 150 nm sees its energetic reservoir and burn rate dropping of 40% and 20%, respectively. The same Al/CuO stack with a bilayer thickness of 1 µm has an energetic reservoir and burn rate dropping of 6% and 16%, respectively. Finally, based on a redox reaction model specifically calibrated on our Al/CuO sputter deposited nanolaminates, we provided temperature/time diagram allowing to perform accelerated aging, which can be of great interest for future applications.

## Figures and Tables

**Table 1:** Model parameters used for aging simulations of Al/CuO nanolaminates. Thermo-physical parameters are given in Supporting Information file, **Table S1**.

Mechanisms	Arrhenius Parameters	Source
Oxygen diffusion through natural Al_x_Cu_y_O_z_	$D_{0}$ = 1 ×$10^{-7}$ m^2^·s^−1^ $E_{a}$ = 100 kJ·mol^−1^	Calibrated from DSC experiments, see Ref. [26]
Oxygen diffusion through amorphous ${\mathrm{Al}}_{2}O_{3}$	$D_{0}$ = 1.67 × $10^{-11}$ m^2^·s^−1^ $E_{a}$ = 120 kJ·mol^−1^	Isotopic labelling, see Ref. [29]

**Table 2:** Interfacial alumina thickness and Energy Loss (EL%) after 30 years of storage at the ambient temperature for 9 sets of Al/CuO nanolaminates (3 stoichiometries and, for each, 3 bilayer thicknesses).

Nanolaminate Configuration	Grown Interfacial Alumina Thickness	Energy Loss in %
ξ 1 Al/CuO 75/150	0.3 nm	0.023%
ξ 1 Al/CuO 100/200		0.017%
ξ 1 Al/CuO 150/300		0.008%
ξ 2 Al/CuO 75/75		0.048%
ξ 2 Al/CuO 100/100		0.036%
ξ 2 Al/CuO 150/150		0.018%
ξ 3 Al/CuO 75/50		0.072%
ξ 3 Al/CuO 100/67		0.054%
ξ 3 Al/CuO 150/100		0.027%

**Table 3:** Experimental burn rates obtained on various sputter-deposited Al/CuO nanothermites produced by our team and comparison with theoretical results obtained with a home-built propagation model as detailed in [32].

	Experiments Macroscopic Burn Rate m/s	From Model [32] m/s
ξ 1 Al/CuO 75/150		
15 bilayers		
As-deposited	4.6 ± 0.5	4.2
Aged 14 days at 200 °C	2.7 ± 0.5	3.0
ξ 2 Al/CuO 150/150		
11 bilayers		
As-deposited	11.7 ± 0.5	4.6
Aged 14 days at 200 °C	5.5 ± 0.5	3.8
ξ 3 Al/CuO 225/150		
9 bilayers		
As-deposited	10.1 ± 0.5	4.7
Aged 14 days at 200 °C	3.3 ± 0.5	3.7

## Supplementary Materials

The following are available online at https://www.mdpi.com/2079-4991/10/10/2087/s1, Table S1: Physical and thermodynamic parameters used in the model.

## References

[1] Bezmelnitsyn A., Thiruvengadathan R., Barizuddin S., Tappmeyer D., Apperson S., Gangopadhyay K., Gangopadhyay K., Redner P., Donadio M., Kapoor D. (2010). Modified Nanoenergetic Composites with Tunable Combustion Characteristics for Propellant Applications. Propellants Explos. Pyrotech..

[2] Staley C.S., Raymond K.E., Thiruvengadathan R., Apperson S.J., Gangopadhyay K., Swaszek S.M., Taylor R.J., Gangopadhyay S. (2013). Fast-Impulse Nanothermite Solid-Propellant Miniaturized Thrusters. J. Propuls. Power.

[3] Zhou X., Torabi M., Lu J., Shen R., Zhang K. (2014). Nanostructured Energetic Composites: Synthesis, Ignition/Combustion Modeling, and Applications. ACS Appl. Mater. Interfaces.

[4] Stamatis D., Dreizin E.L., Higa K. (2011). Thermal Initiation of Al-MoO_3_ Nanocomposite Materials Prepared by Different Methods. J. Propuls. Power.

[5] Duckham A., Spey S.J., Wang J., Reiss M.E., Weihs T., Besnoin E., Knio O.M. (2004). Reactive nanostructured foil used as a heat source for joining titanium. J. Appl. Phys..

[6] Rossi C., Zhang K., Esteve D., Alphonse P., Tailhades P., Vahlas C. (2007). Nanoenergetic Materials for MEMS: A Review. J. Microelectromech. Syst..

[7] Xu J., Shen Y., Wang C., Dai J., Tai Y., Ye Y., Shen R., Wang H., Zachariah M.R. (2019). Controlling the energetic characteristics of micro energy storage device by in situ deposition Al/MoO_3_ nanolaminates with varying internal structure. Chem. Eng. J..

[8] Zhou X., Shen R., Ye Y., Zhu P., Hu Y., Wu L. (2011). Influence of Al/CuO reactive multilayer films additives on exploding foil initiator. J. Appl. Phys..

[9] Zhu P., Shen R., Ye Y., Fu S., Li D. (2013). Characterization of Al/CuO nanoenergetic multilayer films integrated with semiconductor bridge for initiator applications. J. Appl. Phys..

[10] Zhu P., Shen R., Fiadosenka N.N., Ye Y., Hu Y. (2011). Dielectric structure pyrotechnic initiator realized by integrating Ti/CuO-based reactive multilayer films. J. Appl. Phys..

[11] Nicollet A., Lahiner G., Belisario A., Assié-Souleille S., Rouhani M.D., Estève A., Rossi C. (2017). Investigation of Al/CuO multilayered thermite ignition. J. Appl. Phys..

[12] Taton G., Lagrange D., Conedera V., Renaud L., Rossi C. (2013). Micro-chip initiator realized by integrating Al/CuO multilayer nanothermite on polymeric membrane. J. Micromech. Microeng..

[13] Fu S., Shen R., Zhu P., Ye Y. (2019). Metal–interlayer–metal structured initiator containing Al/CuO reactive multilayer films that exhibits improved ignition properties. Sens. Actuators A Phys..

[14] Korampally M., Apperson S.J., Staley C.S., Castorena J.A., Thiruvengadathan R., Gangopadhyay K., Mohan R.R., Ghosh A., Polo-Parada L., Gangopadhyay S. (2012). Transient pressure mediated intranuclear delivery of FITC-Dextran into chicken cardiomyocytes by MEMS-based nanothermite reaction actuator. Sens. Actuators B Chem..

[15] Sullivan K.T., Piekiel N.W., Chowdhury S., Wu C., Zachariah M.R., Johnson C.E. (2010). Ignition and Combustion Characteristics of Nanoscale Al/AgIO_3_: A Potential Energetic Biocidal System. Combust. Sci. Technol..

[16] Sullivan K.T., Wu C., Piekiel N.W., Gaskell K., Zachariah M.R. (2013). Synthesis and reactivity of nano-Ag_2_O as an oxidizer for energetic systems yielding antimicrobial products. Combust. Flame.

[17] Rossi C. (2018). Engineering of Al/CuO Reactive Multilayer Thin Films for Tunable Initiation and Actuation. Propellants Explos. Pyrotech..

[18] Dreizin E.L. (2009). Metal-based reactive nanomaterials. Prog. Energy Combust. Sci..

[19] Egan G.C., Mily E.J., Maria J.-P., Zachariah M.R. (2015). Probing the Reaction Dynamics of Thermite Nanolaminates. J. Phys. Chem. C.

[20] Manesh N.A., Basu S., Kumar R. (2010). Experimental flame speed in multi-layered nano-energetic materials. Combust. Flame.

[21] Mily E., Oni A., Lebeau J., Liu Y., Brown-Shaklee H., Ihlefeld J.F., Maria J.-P. (2014). The role of terminal oxide structure and properties in nanothermite reactions. Thin Solid Films.

[22] Zhu P., Shen R.Q., Ye Y.H., Zhou X., Hu Y., Wu L.Z. Energetic Igniters Based on Al/CuO/B/Ti Reactive Multilayer Films. Proceedings of the 2011 International Autumn Seminar on Propellants, Explosives and Pyrotechnics.

[23] Nie H., Chan H.Y., Pisharath S., Hng H.H. (2020). Combustion characteristic and aging behavior of bimetal thermite powders. Def. Technol..

[24] Wang C.-A., Xu J.-B., Shen Y., Wang Y.-T., Yang T.-L., Zhang Z.-H., Li F.-W., Shen R.-Q., Ye Y. (2020). Thermodynamics and performance of Al/CuO nanothermite with different storage time. Def. Technol..

[25] Abdallah I., Zapata J., Lahiner G., Warot-Fonrose B., Cure J., Chabal Y.J., Esteve A., Rossi C. (2018). Structure and Chemical Characterization at the Atomic Level of Reactions in Al/CuO Multilayers. ACS Appl. Energy Mater..

[26] Lahiner G., Zapata J., Cure J., Richard N., Djafari-Rouhani M., Estève A., Rossi C. (2019). A redox reaction model for self-heating and aging prediction of Al/CuO multilayers. Combust. Theory Model..

[27] Deal B.E., Grove A.S. (1965). General Relationship for the Thermal Oxidation of Silicon. J. Appl. Phys..

[28] Fischer S., Grubelich M. Theoretical energy release of thermites, intermetallics, and combustible metals. Proceedings of the Twenty-Fourth International Pyrotechnics Seminar.

[29] Nabatame T., Yasuda T., Nishizawa M., Ikeda M., Horikawa T., Toriumi A. (2003). Comparative studies on oxygen diffusion coefficients for amorphous and gamma-Al_2_O_3_ films using O-18 isotope. Jpn. J. Appl. Phys..

[30] Julien B., Cure J., Salvagnac L., Josse C., Esteve A., Rossi C. (2020). Integration of Gold Nanoparticles to Modulate the Ignitability of Nanothermite Films. ACS Appl. Nano Mater..

[31] Zapata J., Nicollet A., Julien B., Lahiner G., Esteve A., Rossi C. (2019). Self-propagating combustion of sputter-deposited Al/CuO nanolaminates. Combust. Flame.

[32] Lahiner G., Nicollet A., Zapata J., Marín L., Richard N., Rouhani M.D., Rossi C., Estève A. (2017). A diffusion–reaction scheme for modeling ignition and self-propagating reactions in Al/CuO multilayered thin films. J. Appl. Phys..

[33] Wang H., Julien B., Kline D., Alibay Z., Rehwoldt M.C., Rossi C., Zachariah M.R. (2020). Probing the Reaction Zone of Nanolaminates at ∼μs Time and ∼μm Spatial Resolution. J. Phys. Chem. C.

