# Supplementary material for: How Thermal Aging Affects Ignition and Combustion Properties of Reactive Al/CuO Nanolaminates: A Joint Theoretical/Experimental Study
Source: Nanomaterials (Basel). 2020 Oct 21;10(10):2087. doi: 10.3390/nano10102087 (PMC7589912; doi:10.3390/nano10102087)
Supplement: Supplementary file 1 [file nanomaterials-10-02087-s001.pdf]

# Supporting Information

# How Thermal Aging Affects Ignition and Combustion Properties of Reactive Al/CuO Nanolaminates: A Joint Theoretical/Experimental Study

**A. Estève [1], G. Lahiner [1], B. Julien [1], S. Vivies [1], N. Richard [2] and C. Rossi [1,*]**

[1] LAAS-CNRS, University of Toulouse, 7 Avenue du colonel Roche, 31077 Toulouse, France; aesteve@laas.fr (A.E.); glahiner@laas.fr (G.L.); bjulien@laas.fr (B.J.); svivies@laas.fr (S.V.)
[2] CEA-DAM, DIF, 91297 Arpajon, France; nicolas.richard@cea.fr
* Correspondence: rossi@laas.fr

**Table S1: Physical and thermodynamic parameters used in the model**

The enthalpies and temperatures associated to the Al+CuO chemical phase transitions, as well as the melting and boiling of all species, are extracted from the CRC Handbook of Chemistry and Physics [1] and reported here below. We consider these literature values, corresponding to solid/gas equilibrium contexts, since there is no data relative to thin films. The equations describing the specific heat as a function of the temperature for each species come from the NIST Chemistry WebBook: http://webbook.nist.gov/chemistry/.

| | **Al** | **CuO** | **$Cu_2O$** | **Cu** | **$Al_2O_3$** |
|---|---|---|---|---|---|
| **Density (kg.m$^{-3}$)** | 2698 | 6313 | 5983 | 8960 | 3970 |
| **Molar mass (g.mol$^{-1}$)** | 26.98 | 79.54 | 143.08 | 63.54 | 101.96 |
| **Molar enthalpy of formation (kJ.mol$^{-1}$)** | 0 | −157.3 | −168.6 | 0 | −1675.7 |
| **Thermal conductivity (W.m$^{-1}$.K$^{-1}$)** | 237 | 0.847 | 1 | 401 | 10 |
| **Heat of fusion (kJ.mol$^{-1}$)** | 10.79 | 17.47 | 17.47 | 13 | 111 |
| **Melting point (°C)** | 660 | 1326 | 1232 | 1085 | 2054 |
| **Boiling point (°C)** | 2470 | 1997 | 1797 | 2562 | 2977 |

[1] W.M. Haynes, D.R. Lide, T.J. Bruno, CRC handbook of chemistry and physics: a ready-reference book of chemical and physical data., CRC Press, 2017.
